# Human splicing diversity and the extent of unannotated splice junctions across human RNA-seq samples on the Sequence Read Archive

**DOI:** 10.1186/s13059-016-1118-6

**Published:** 2016-12-30

**Authors:** Abhinav Nellore, Andrew E. Jaffe, Jean-Philippe Fortin, José Alquicira-Hernández, Leonardo Collado-Torres, Siruo Wang, Robert A. Phillips III, Nishika Karbhari, Kasper D. Hansen, Ben Langmead, Jeffrey T. Leek

**Affiliations:** 1 Department of Computer Science, Johns Hopkins University, Baltimore, MD USA; 2 Department of Biostatistics, Johns Hopkins Bloomberg School of Public Health, Baltimore, MD USA; 3 Center for Computational Biology, Johns Hopkins University, Baltimore, MD USA; 4 Lieber Institute for Brain Development, Johns Hopkins Medical Campus, Baltimore, MD USA; 5 Department of Mental Health, Johns Hopkins University, Baltimore, MD USA; 6 Undergraduate Program in Genome Sciences, National Autonomous University of Mexico, Mexico City, Mexico; 7 Department of Mathematics and Computer Science, Centre College, Danville, KY USA; 8 Department of Biological Sciences, Salisbury University, Salisbury, MD USA; 9 Department of Biological Sciences, University of Texas at Austin, Austin, TX USA; 10 McKusick-Nathans Institute of Genetic Medicine, Johns Hopkins University, Baltimore, MD USA

**Keywords:** RNA-seq, Splicing, Intron

## Abstract

**Background:**

Gene annotations, such as those in GENCODE, are derived primarily from alignments of spliced cDNA sequences and protein sequences. The impact of RNA-seq data on annotation has been confined to major projects like ENCODE and Illumina Body Map 2.0.

**Results:**

We aligned 21,504 Illumina-sequenced human RNA-seq samples from the Sequence Read Archive (SRA) to the human genome and compared detected exon-exon junctions with junctions in several recent gene annotations. We found 56,861 junctions (18.6%) in at least 1000 samples that were not annotated, and their expression associated with tissue type. Junctions well expressed in individual samples tended to be annotated. Newer samples contributed few novel well-supported junctions, with the vast majority of detected junctions present in samples before 2013. We compiled junction data into a resource called intropolis available at http://intropolis.rail.bio. We used this resource to search for a recently validated isoform of the ALK gene and characterized the potential functional implications of unannotated junctions with publicly available TRAP-seq data.

**Conclusions:**

Considering only the variation contained in annotation may suffice if an investigator is interested only in well-expressed transcript isoforms. However, genes that are not generally well expressed and nonetheless present in a small but significant number of samples in the SRA are likelier to be incompletely annotated. The rate at which evidence for novel junctions has been added to the SRA has tapered dramatically, even to the point of an asymptote. Now is perhaps an appropriate time to update incomplete annotations to include splicing present in the now-stable snapshot provided by the SRA.

## Background

Gene annotations such as those compiled by RefSeq [1] and GENCODE [2] are derived primarily from alignments of spliced complementary DNA (cDNA) sequences and protein sequences [3, 4]. So far, the impact of RNA sequencing (RNA-seq) data on annotation has been limited to a few projects including ENCODE [5] and Illumina Body Map 2.0 [6].

To measure how much splicing variation present in publicly available RNA-seq datasets is missed by annotation, we aligned 21,504 Illumina-sequenced human RNA-seq samples from the Sequence Read Archive (SRA) to the *hg19* genome assembly with Rail-RNA [7] and compared exon-exon junction calls to exon-exon junctions from annotated transcripts. We compared exon-exon junctions rather than full transcripts because junction calls from short RNA-seq reads are considerably more reliable than assembled transcripts [8]. Details of our alignment protocol are reviewed in Methods. All alignment was performed in the cloud using Amazon Web Services (AWS) Elastic MapReduce, costing 72 US cents per sample, as computed in Methods.

We considered only Illumina platforms because of their ubiquity and high base-calling accuracy. Specifically, the samples we aligned were obtained by querying the SRA metadata SQLite database of the R/Bioconductor package SRAdb [9] as of April 2015 for all Illumina-sequenced human RNA-seq samples.

In the remainder of this paper, we use the term “annotation” to refer to junctions from the union of transcripts across several gene annotation tracks from the UCSC Genome Browser [10]. We also occasionally use the term “RNA-seq junctions” to distinguish junctions we called from RNA-seq data from annotated junctions. For *hg38* annotations, coordinates were lifted over to *hg19*. See Methods for details and Table 1 for included gene annotations together with the number of junctions in each. In all, we found 536,994 annotated junctions in RNA-seq data: 505,314 were present in annotations of *hg19*, and the rest were added by annotations of *hg38*.

**Table 1:** Gene annotations from which exon-exon junctions were extracted and unioned to obtain a list of annotated junctions

Gene annotation	Number of junctions	Reference
*hg19*		
AceView	395,410	[12]
CCDS	174,337	[43]
GENCODE v19	343,887	[2]
UCSC	264,339	[10]
lincRNAs	30,256	[6]
MGC	160,388	[44]
RefSeq	235,512	[1]
SIB	414,422	[45]
Vega	242,672	[2]
*hg38*		
CCDS	178,775	[43]
GENCODE v24	346,547	[2]
UCSC	356,644	[10]
lincRNAs	29,940	[6]
MGC	169,302	[44]
RefSeq	247,577	[1]
SIB	433,606	[45]

All tracks were taken from the UCSC Genome Browser [10] except for GENCODE [2], which was downloaded from the GENCODE website http://www.gencodegenes.org/releases/. Junction coordinates from *hg38* annotations were lifted over to *hg19* before the union was performed. Of all gene annotations listed here, the Swiss Institute of Bioinformatics (SIB) genes has the most, with more than 400,000 junctions for each of *hg19* and *hg38*

## Results and discussion

We compiled the junction calls and associated coverage levels for 21,504 SRA RNA-seq samples into a resource called intropolis available at http://intropolis.rail.bio. Using this resource, we addressed several questions that are fundamental to our understanding of the transcriptome and informative for analyses by individual investigators.

### Reproducibility of junction calls across alignment protocols

**Fig. 1:**
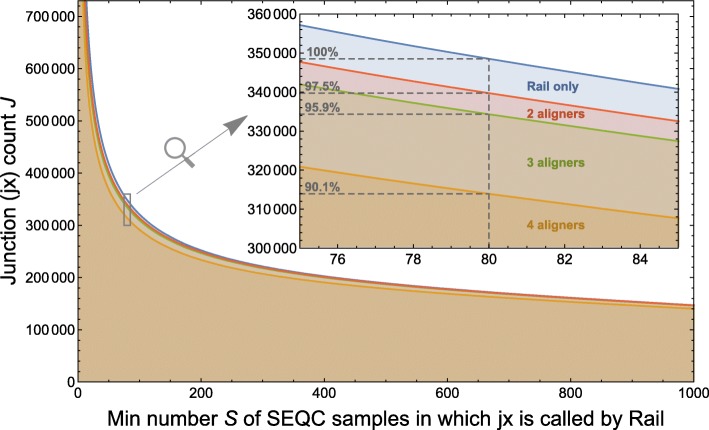
Displayed is the number of exon-exon junctions *J* found by Rail-RNA and other alignment protocols in at least *S* of the 1720 brain and universal human reference RNA-seq samples also studied by the SEQC/MACQ-III consortium [11] (i.e., SEQC). “2 aligners” (*red*), “3 aligners” (*green*), and “4 aligners” (*orange*) refer to junctions we found with Rail-RNA that were also found by, respectively, 1, 2, and 3 of the alignment protocols used by SEQC

We first asked whether our RNA-seq junction calls could be reproduced across alignment protocols. The SEQC/MACQ-III consortium (hereafter called SEQC) aligned a subset of 1720 universal human reference RNA and human brain reference RNA samples [11] of the 21,504 samples we considered using three different protocols: NCBI Magic [12], r-make (which uses STAR [13]) and Subread [14]. Junctions called by Rail-RNA are compared with junctions called by SEQC across the subset in Fig. 1. Of junctions found by Rail-RNA in at least 80 SEQC samples, as many as 97.5% are found by at least one SEQC alignment protocol, and 90.1% are found by all three. Note that 80 SEQC samples is 4.7% of 1720, comparable to a 1000-sample threshold discussed in the next subsection for the 21,504 SRA samples. This suggests that the overwhelming majority of junctions we called are not artifacts of any particular aligner’s junction-calling algorithm.

### Relationship between annotation and expression of splice junctions

We next asked whether annotated junctions represent the diversity of junction expression observed in the population at large. We considered an RNA-seq junction to be well supported in our data if it was observed in a large number of samples. We calculated the number of junctions that appeared in at least *S* samples across a range of cutoffs. For each RNA-seq junction we considered, we also evaluated whether it appeared in annotation. We considered the following levels of evidence: (1) fully annotated junctions; (2) separately annotated junctions (typically exon-skipping events), where both the donor and acceptor sites appear in one or more junctions from annotation, but never for the same junction; (3) alternative donor and acceptor sites, where only either the donor or the acceptor site appears in one or more junctions from annotation; and (4) novel junctions, where neither donor nor acceptor site is found in any annotated junction.

**Fig. 2:**
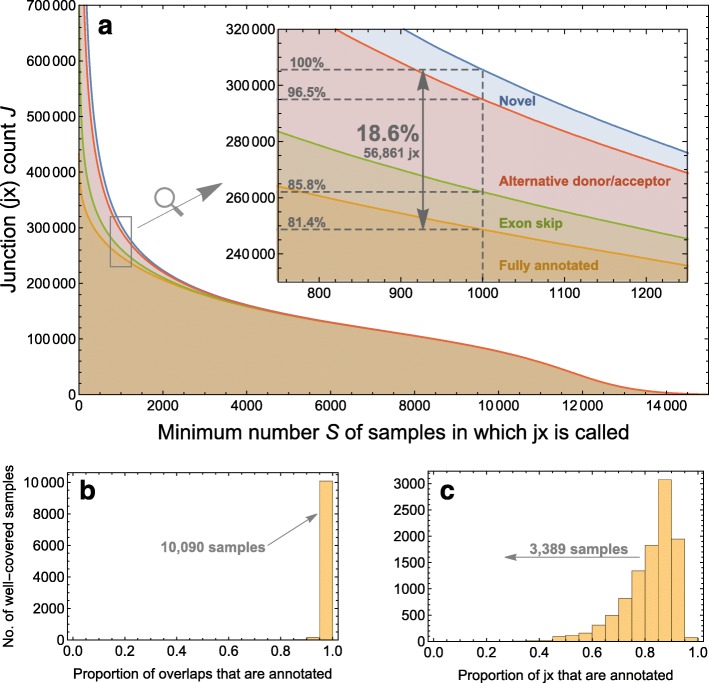
**a** Shows how many exon-exon junctions *J* are found in at least *S* samples of the 21,504 human RNA-seq samples on the SRA aligned here. It also shows how much evidence for these junctions is found in gene annotation: “fully annotated” (*orange*) means the junction is in an annotated transcript, “exon skip” (*green*) means a called junction’s donor and acceptor sites are annotated in distinct junctions, “alternative donor/acceptor” (*red*) means only one of a called junction’s donor and acceptor sites is in a junction from annotation, and “novel” (*blue*) means neither donor nor acceptor site is annotated. **b** and **c** restrict attention to the 10,311 samples for which 100,000 junctions are discovered in each. **b** refers to overlaps, where an overlap is any instance where a read maps across a junction

We observed that the RNA-seq junctions most widely expressed across samples and experiments were well documented in annotation. For example, we observed that junctions that appeared in at least 40% of human RNA-seq samples on the SRA (*S*≥8000) were also present in previous annotation at least 99.8% of the time. However, 18.6% of junctions that appeared in 1000 or more samples did not appear in annotation (Fig. 2 a). Many of these unannotated junctions are partially annotated, but 3.5% of junctions found in more than 1000 samples do not match any splice site from an annotated junction.

We also took an investigator-focused view of the relationship between annotation and expression. Most investigators collect only a small number of samples for their study. We restricted attention to samples where at least 100,000 RNA-seq junctions were found to rule out obviously small RNA-seq samples and samples that were mislabeled as RNA-seq on the SRA. In each sample, we counted the number of instances where a read maps across a junction. (A read mapping across two junctions thus contributes two instances.) The total number of such “junction overlaps” across samples is a measure of the total expression of junctions across the SRA. Most of the reads that map to junctions map to annotated junctions (Fig. 2 b). In 10,090 of a total of 10,311 samples that meet our criterion of 100,000 junctions observed, more than 95% of junction overlaps correspond to annotated junctions.

This represents only the bulk coverage of junctions. We can also consider the number of junctions observed, regardless of coverage. In 3389 out of 10,311 samples, we observe that fewer than 80% of junctions appear in annotation (Fig. 2 c). So while the most highly covered junctions are well annotated, there is a large number of junctions that are well covered across multiple samples but may not appear in any given small subset of samples.

**Fig. 3:**
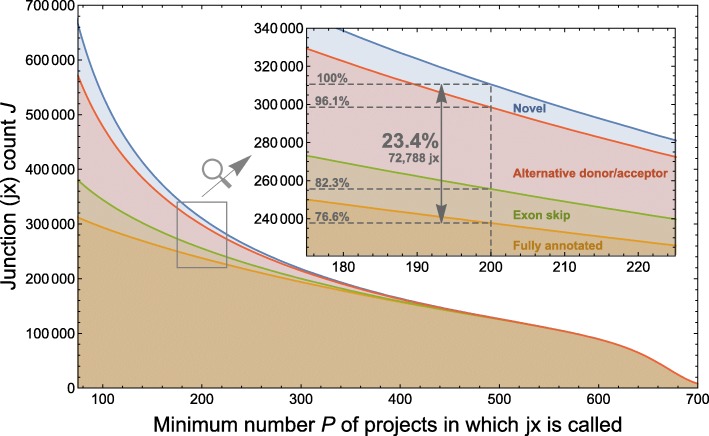
Displayed is the number of exon-exon junctions *J* found in at least *P* projects of the 929 human RNA-seq projects on the SRA considered in this paper. It also shows how much evidence for these junctions is found in gene annotation: “fully annotated” (*orange*) means the junction is in an annotated transcript, “exon skip” (*green*) means a called junction’s donor and acceptor sites are annotated in distinct junctions, “alternative donor/acceptor” (*red*) means only one of a called junction’s donor and acceptor sites is in a junction from annotation, and “novel” (*blue*) means neither donor nor acceptor site is annotated

To explore this idea further, we investigated the potential for single studies to be the sole contributors of individual unannotated junctions. In this event, the junction may not have been called robustly across experimental protocols. Here, we considered junctions that appeared in at least *P* projects instead of samples. We again broke this calculation down by the different potential levels of evidence: whether the junction was entirely novel, had an alternative donor or acceptor, an exon skip, or whether it was fully annotated (Fig. 3). The story at the project level mirrors the story at the sample level: 23.4% of junctions found in more than 200 of the 929 projects are not fully annotated. So unannotated junctions recur across independent investigations.

### Technical and biological variation in junction expression across samples

We next explored variation across the 21,504 samples we processed. We wanted to see the combination of technical and biological factors that contribute to variation in unannotated junction expression. In this analysis, we considered only the 56,861 unannotated junctions found in at least 1000 samples of the 21,504, and the subset of 21,057 samples of the 21,504 with at least 100,000 reads each. We performed a principal component analysis (PCA) on the data matrix where rows correspond to the 56,861 unannotated junctions and columns correspond to the 21,057 samples. (See Methods for technical details of the decomposition.)

**Fig. 4:**
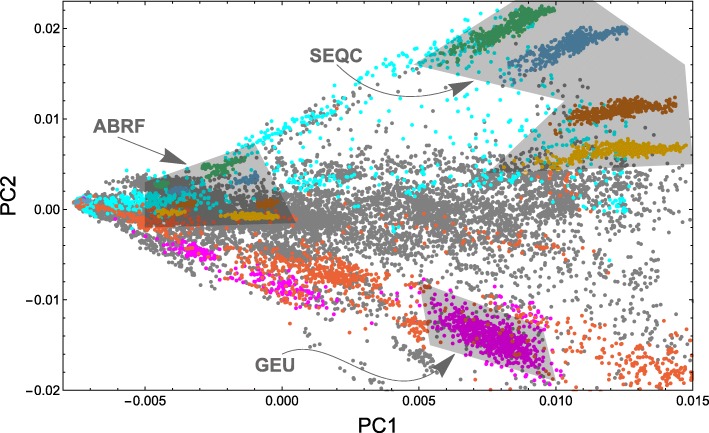
Displayed is the first principal component (*PC1*) vs. the second principal component (*PC2*) for a principal component analysis (*PCA*) with a coverage data matrix where rows are junctions and columns are samples. (See Methods for technical details.) Each point corresponds to a distinct sample. *Gray* points are unlabeled samples, *red* points are blood samples, *magenta* points are lymphoblastoid cell line samples, and *cyan* points are brain samples. GEUVADIS (*GEU*) is a sizable cluster of magenta points. The *ABRF* and *SEQC* consortia each sequenced mixtures of universal human reference RNA (*UHRR*) and human brain reference RNA (*HBRR*) in four sample ratios UHRR:HBRR that form distinct clusters in the shaded regions: 0:1 (*green*), 1:3 (*blue*), 3:1 (*brown*), and 1:0 (*yellow*)

PC1 explains the overwhelming majority of the variance (87.9%) and has a Pearson correlation coefficient *r*=0.978 with junction sequencing depth *s*_*j*_ as measured by total junction overlaps (i.e., instances where a read maps across a junction) in sample *j* (Fig. 4) after normalization by library size and log transformation. PC1 is also highly correlated with log-transformed read length *ℓ*_*j*_ (*r*=0.639), but much less correlated with log-transformed total number of mapped reads *C*_*j*_ (*r*=0.277), showing that enrichment for splice junctions is different in different samples. (See Methods for precise definitions of correlates.)

We further examined samples belonging to specific groups that generated well-characterized datasets. Both the SEQC consortium and ABRF [15] studied universal human reference RNA (UHRR) and human brain RNA reference (HBRR) samples constructed by the MACQ-III consortium for quality control. UHRR comprises total RNA from ten different cancer cell lines representing various human tissues, while HBRR samples comprise total RNA from several donors across several brain regions. Both groups studied these samples in four different mixture ratios—0:1, 1:3, 3:1, and 1:0—with each sample sequenced at multiple sites. The four mixtures separate well, and each lies on a radial line passing through the singular point on the left. Data from the two groups are separated because they used different sequencing depths and read lengths.

The four SEQC UHRR:HBRR sample ratios form four clusters distinguished by PC2, and the ABRF UHRR:HBRR sample ratios form clusters distinguished by both PC1 and PC2. Observe that there is a singular point where all points appear to converge (Fig. 4). Here, the number of junctions detected in a sample approaches zero. A radial line extending from the singular point rotating clockwise across the plot passes over UHRR:HBRR sample ratios in the same order for ABRF as it does for SEQC. Though ABRF and SEQC have some overlap in managing investigators, they are two different projects that employed randomized study designs, making a strong case that PC2 is distinguishing mostly biological rather than technical factors.

Lymphoblastoid cell lines, typically made from HapMap samples, are extensively present in the SRA. Different studies cluster together and are again placed on a radial line going through the singular point; each study used very different sequencing depths and read lengths. Searching the SRA metadata, we could classify a number of samples as brain and blood. Again, these samples fall along radial lines through the singular point. The biggest separation in PC2 is between brain and blood, two tissue types that are well represented in the SRA.

### Novel junction discovery over time

We proceeded to measure the accumulation of “confidently called” junctions over calendar time. A junction was “confidently called” if it was found in at least 20 reads across all samples. We measured the discovery date of a junction as the earliest submission date to the BioSample database [16] from among all samples in which the junction was found by Rail-RNA. The ≥20-read curve has noticeable spikes in 2009 and 2011 but appears to decelerate significantly before 2013, by which time 96.1% of junctions were discovered.

**Fig. 5:**
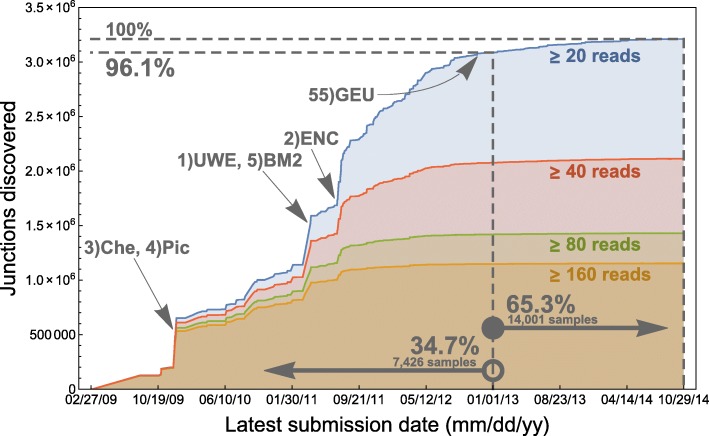
The 3,211,228 junctions found in at least 20 reads across samples are accumulated by their “discovery dates.” Here, discovery date of a junction is taken to be the earliest submission date to the BioSample database from among the samples in which the junction was found. 96.1% of the junctions were discovered before 1 January 2013, although only 34.7% of samples depicted in the figure had been submitted by then, and afterwards discovery levels off. Demanding higher levels of confidence (the *red, green,* and *orange curves*) gives rise to earlier asymptotes. Ranked from 1 to 5 are the dominant contributing projects from dates on which the most junctions are discovered. “Che” refers to a study of 41 Coriell cell lines by Cheung et al. [21], “Pic” refers to a study of 69 LCLs by Pickrell et al. [20], “UWE” refers to the University of Washington Human Reference Epigenome Mapping Project [17], “BM2” refers to Illumina Body Map 2.0 [6], and “ENC” refers to ENCODE [19]. “GEU” refers to GEUVADIS [37], whose 464 LCLs uncovered few junctions that had not already been discovered

Recent samples added to the SRA have contributed few novel junctions. Curves for more stringent coverage thresholds (Fig. 5) level off sooner; the curve for the most stringent threshold (≥160 reads) is essentially flat by 2012. Ranked and labeled are the dominant contributing projects from days on which the most junctions were discovered. The largest single contribution comes from UWE, the University of Washington’s Human Reference Epigenome Mapping Project [17], on 4 April 2011, when 252,628 new junctions appeared. The submission includes total RNA from fetal tissue, which exhibits markedly different expression than adult tissue [18]. Moreover, sequencing total RNA may capture a more diverse range of exon-exon junction expression than sequencing poly(A)-selected RNA since a larger set of noncoding transcripts is sampled. So a new total RNA sample may contribute more novel junctions than a new poly(A)-selected sample. The second, third, fourth, and fifth largest contributions are from, respectively, ENCODE [19], early studies of 69 lymphoblastoid cell lines (LCLs) [20] and 41 Coriell cell lines [21], and the Illumina Body Map 2.0 sequencing of 16 human tissue types [6]. The GEUVADIS submission of 464 LCLs is on only the 55th largest contributing date, 7 November 2012. By this time, LCLs had already been well studied using RNA-seq.

**Fig. 6:**
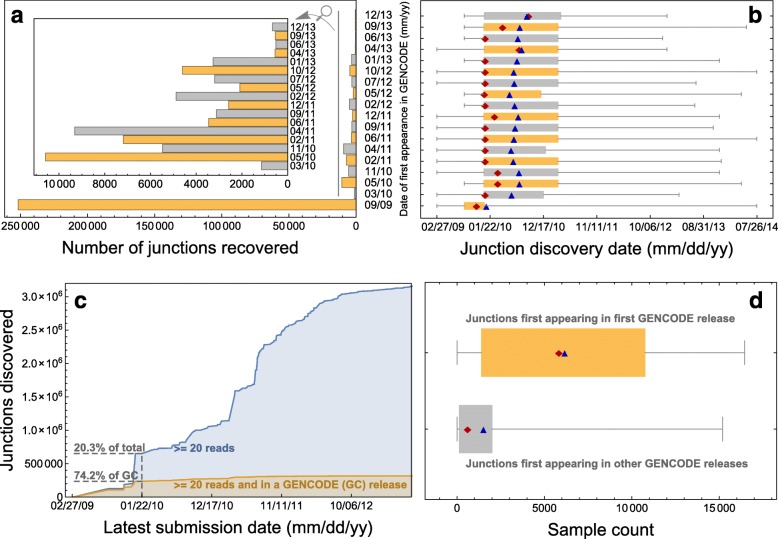
Displayed is a summary of the evolution of junctions from the GENCODE annotation of *hg19* through its 18 releases compared to the evolution of confidently called junctions called across the SRA. Every junction considered here is “confidently called”—found in at least 20 reads across the SRA samples we analyzed. **a** shows that most junctions (80.0%) annotated by GENCODE first appeared in the first release. **b** shows that junctions in GENCODE tend to have early discovery dates. This is also evident from **c**, which shows that while only 20.3% of junctions are discovered by late January 2010, almost three-quarters of junctions appearing in at least one GENCODE release are discovered by the same date. Also shown in **b** is how junctions first appearing in GENCODE’s first release have noticeably earlier discovery dates than junctions first appearing in later releases. This is due to how junctions first appearing in GENCODE’s first release tend to be found in many more samples (median = 5825) than junctions first appearing in later releases (median = 602 samples), as shown in **d**. In every box plot, the *red diamond* corresponds to the median, and the *blue triangle* corresponds to the mean

To determine whether the annotation of junctions is being driven by RNA-seq experiments, we examined the correlation between annotated junctions and the discovery date of observed junctions over calendar time. GENCODE released 18 versions between September 2009 and December 2012. Call a confidently called junction “documented” if it appears in at least one GENCODE release. Most documented junctions (80.0%) appear in the earliest GENCODE release (Fig. 6 a). Documented junctions tend to have early discovery dates (Fig. 6 b); in fact, by late January 2010, 74.2% of documented junctions were discovered, while 20.3% of confidently called junctions were discovered (Fig. 6 c). This makes sense: annotated junctions tend to be found in many samples, making it likelier that at least one sample has an early submission date to BioSample. It is reasonable to speculate that there is a correlation between junction discovery date and GENCODE appearance date: perhaps shortly after a junction is discovered, it appears in GENCODE.

But inspection of the relationship between documentation date and discovery date suggests that only the first GENCODE release introduced new junctions with significantly earlier discovery dates than other releases (Fig. 6 b). The reason for this phenomenon is that junctions appearing first in GENCODE’s first release are present in many more samples (median = 5825) than junctions appearing first in other GENCODE releases (median = 602 samples) (Fig. 6 d).

### Application to *ALK* isoform discovery

We have compared the variation in our database intropolis to standard gene annotations.

intropolis associates each junction with the set of samples where the junction was called and the number of reads spanning the junction in that sample, enabling biological investigators to gain new insights. Here, we give a simple example application involving the anaplastic lymphoma kinase (*ALK*) gene.

*ALK* is frequently mutated or aberrantly expressed in cancers including neuroblastoma [22–25] and non-small-cell lung adenocarcinoma, where in particular it has been found to participate in the fusion gene *EML4-ALK* [26]. Cancers with *ALK* abnormalities are often responsive to treatment with *ALK* inhibitors such as crizotinib [27]. *ALK* is a good therapeutic target because it is rarely expressed in normal adult tissue [28]. A novel *ALK* transcript variant present in about 11% of melanomas and occasionally in other cancer subtypes was recently identified [29]. The transcript is described as resulting from a *de novo* alternative transcription initiation (ATI) site in *ALK* intron 19 and is dubbed *ALK* ^*ATI*^. The kinase activity of *ALK* ^*ATI*^ is found to be suppressed by various *ALK* inhibitors, and a patient with *ALK* ^*ATI*^-expressing metastatic melanoma is shown to exhibit significant tumor shrinkage after treatment with crizotinib.

**Fig. 7:**
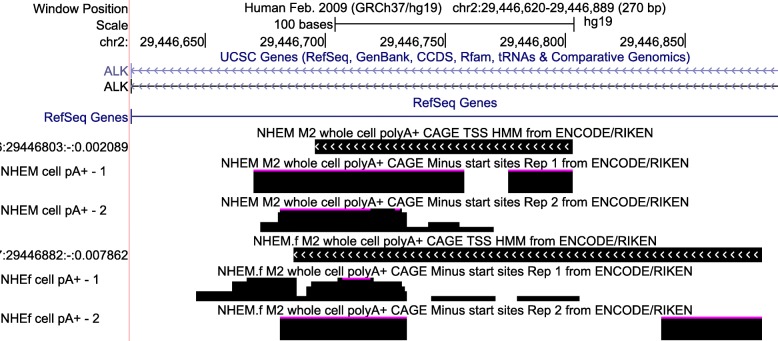
Displayed in the UCSC Genome Browser (http://genome.ucsc.edu) are tracks corresponding to CAGE data for normal human melanocyte cell cultures NHEM_M2 and NHEM.f_M2 studied by ENCODE as well as TSSes predicted with hidden Markov models from pooled replicates in the *ALK* gene for *hg19*. Observe that one model predicts a TSS in the region chr2:29,446,803–29,446,696 and the other predicts a TSS in the region chr2:29,446,882–29,446,687, both of which contain the TSS region identified for *ALK* ^*ATI*^ in [29], chr2:29,446,768–29,446,744

To investigate the prevalence of *ALK* ^*ATI*^ on the SRA, we searched for a deficit of junction expression in *ALK* exons 1–19 compared to exons 20–29. We did this by defining a junction inclusion ratio *D* measuring to what degree junctions between exons 20–29 are expressed relative to junctions between exons 1–19 (see Methods). This signature is a necessary but not sufficient condition for exclusive *ALK* ^*ATI*^ expression: the expression signature also arises in, for example, the *EML4-ALK* fusion gene. Table 2 shows the ten top SRA samples we studied ranked in order of decreasing *D*. As expected, four such samples are cancers, including uveal melanoma. Three of the ten samples are from two melanocyte cell cultures studied as part of the ENCODE project, “NHEM_M2” and “NHEM.f_M2.” Cap analysis of gene expression (CAGE) data from ENCODE on the same cell lines shows a transcription start site (TSS) within *ALK* intron 19, where the TSS was localized for *ALK* ^*ATI*^ (Fig. 7). This raises the possibility that the transcript is expressed in normal melanocytes. While [29] found no *ALK* ^*ATI*^ expression in 1600 samples from 43 different normal tissues across the GTEx project, including skin, it should be noted that melanocytes comprise only up to 10% of skin cells. In addition to melanocytes, the *ALK* ^*ATI*^ transcript may be expressed in macrophages. We also observed that the macrophage and macrophage+fibroblast samples from Table 2 are part of the study [30] that additionally sequenced the same samples exposed to tumor necrosis factor (TNF). The two samples exposed to TNF appear to have no expression of the *ALK* gene, suggesting that TNF may participate in suppressing *ALK* gene expression. This is supported by [31] in lymphoma.

**Table 2:** Top ten samples across the 21,504 analyzed in this paper in order of descending junction inclusion ratio *D*, as defined in the table

Rank	Sample (i.e., run)	Project	Description of sample	Junction	Junction	Total junction	D=(B-A)/C
				coverage A for ALK	coverage B for ALK	coverage C	
				exons 1–19	exons 20–29	across ALK	
1	SRR545713	SRP007461	NHEM.f_M2: normal human	0	139	139	1
			melanocyte				
1	SRR396804	SRP010166	Non-small cell lung	0	172	172	1
			adenocarcinoma				
1	SRR620100	SRP017262	Leukemia	0	108	108	1
4	SRR1289650	SRP042031	Macrophage	1	85	86	0.976
5	SRR1289651	SRP042031	Macrophage cultured	1	77	78	0.974
			with fibroblast				
6	SRR545716	SRP007461	NHEM_M2: normal human	2	94	96	0.958
			melanocyte				
7	SRR628586	SRP017413	Uveal melanoma	12	111	123	0.805
8	DRR016705	DRP001919	H2228, an EML4-ALK-expressing	38	285	333	0.765
			lung adenocarcinoma cell line				
9	SRR545714	SRP007461	NHEM.f_M2: normal human	14	63	77	0.636
			melanocyte				
10	ERR532612	ERP006077	Prostate tumor	16	53	69	0.536

*D* essentially measures the difference in expression between junctions across *ALK* exons 1–19 and junctions across *ALK* exons 20–29. Values of *D* close to 1 may point toward expression of ALK ^*ATI*^, a novel transcript variant of *ALK* recently identified in [29] across several cancers but not normal cells. Several cancer samples appear, but interestingly, normal cell samples also appear, including melanocytes and macrophages

### Potential functional implications of previously unannotated junctions

We lastly searched for evidence that unannotated and partially annotated junctions were functionally relevant. In [32], Hupe et al. performed translating ribosome affinity purification followed by RNA sequencing (TRAP-seq) of brain and kidney samples from mouse. TRAP is a technology that isolates translating RNAs from intact tissues, potentially from targeted cell types. Thus, we examined the extent of our novel and partially annotated junctions presumably being translated. We aligned the six kidney and nine brain TRAP-seq samples from their study using Rail-RNA and lifted the resulting exon-exon junction coordinates over from mouse (*mm10*) to human (*hg19*) (see Methods). Of the 112,825 junctions found across the TRAP-seq samples whose liftovers were also found in at least one SRA sample, 86,954 (77.1%) were fully annotated, 10,771 (9.5%) were exon skips, 12,410 (11.0%) had alternative donors or acceptors, and 2690 (2.4%) were novel. These data suggest that a significant fraction of unannotated junctions are likely conserved across species: more than 3% of unannotated junctions found in more than 1000 SRA samples have analogs likely translated in mouse. Furthermore, of the 84,185 junctions found across the TRAP-seq samples whose liftovers were also found in at least 1000 SRA samples, 81,482 (96.9%) were fully annotated, 1089 (1.3%) were exon skips, 1464 (1.7%) had alternative donors or acceptors, and 150 (0.2%) were novel. So there is significant evidence that many previously unannotated or partially annotated junctions are translated into proteins and therefore have potentially novel functional relevance.

## Conclusions

We have measured variation in junction expression across thousands of RNA sequencing samples. Our analysis demonstrates both the strengths and weaknesses of relying on current annotation for RNA-seq analysis. We have also used our population-level view of transcription to understand the potential hazards of analyzing individual samples without a clear understanding of the background variation in junction discovery levels. We have shown evidence that some unannotated and partially annotated junctions in human have translated analogs in mouse. We have introduced a resource, intropolis, for others to investigate junction variation, and we have provided an example of the utility of our resource in the case of *ALK* gene expression.

While we observed many unannotated junctions shared by thousands of RNA-seq samples from independent investigations, some of these are likely false positive calls due to incorrect placement of reads, sample-specific variation, and regions where the reference genome is incorrectly assembled. Rail-RNA [7] is designed to be parsimonious and conservative, and its junction calls agree closely with those of other aligners (Fig. 1). When an intropolis junction appears in many samples, our evidence suggests that the call is reliable; e.g., 99.8% of junctions found in at least 8000 samples from the SRA are also found in gene annotation. That said, individual novel junctions in intropolis should be used carefully and verified by other means, such as rtPCR, where appropriate.

Our study also suggests that the rate at which evidence for novel junctions has been added to the SRA has tapered dramatically, even to the point of an asymptote (Fig. 5). This has implications for projects and tools that use gene annotations; if annotations have been incomplete up to this point, now is perhaps an appropriate time to update them to include splicing present in the now-stable snapshot provided by the SRA.

As highlighted by Fig. 2 a, b, considering only the variation contained in annotation may suffice if an investigator is interested only in well-expressed transcript isoforms. However, genes that are not generally well expressed and nonetheless are present in a small but significant number of samples in the SRA are likelier to be incompletely annotated. Quantification of poorly expressed genes may thus be improved by incorporating information about annotated and unannotated splicing events. Along related lines, [33] develops a local splicing variation (LSV) formalism that jointly analyzes multiple junctions from the same gene using splicing graphs. The authors show a significant impact of considering novel (unannotated) junctions on their conclusions. Using this, or similar methodology, on the intropolis database to go beyond the single-junction analysis presented here may be an interesting avenue for future research.

Our approach to synthesizing large public RNA-sequencing datasets offers the opportunity to study transcription more deeply than ever before. Further, intropolis is a step toward establishing public resources that facilitate rapidly querying existing RNA-seq data.

## Methods

### Identifying annotated junctions

Following [34], we extracted junctions from transcripts across all the latest “empirical” gene annotation tracks in the UCSC Genome Browser [10] for *hg19* and *hg38* except GENCODE [2] and Ensembl [35]. (While GENCODE’s tracks are also in the UCSC Genome Browser, we chose to download them from the GENCODE website http://www.gencodegenes.org/releases/ instead: as of 24 January 2016, GENCODE v22 was the latest GENCODE track listed, but GENCODE v24 had already been released.) Empirical tracks are based on alignments of, e.g., spliced cDNA and protein sequences and are listed in Table 1. Annotation tracks based on algorithmic predictions from genome sequence (Augustus, GeneID, Genscan, N-SCAN, and SGP) were excluded because they comprise transcripts that were not directly observed in experiment. Ensembl was excluded because GENCODE is already a merge of Ensembl and HAVANA transcripts. After junction coordinates were extracted, all *hg38* coordinates were lifted over to *hg19* where feasible, and the union of all junctions was taken. Liftover of junctions was performed using the UCSC liftOver utility [36] with command-line parameters -ends 2 -minMatch=1.0. Since the intropolis database was formed from alignments to only the *hg19* chromosomal assembly, only those junctions corresponding to the *hg19* chromosomal assembly were kept to form a final list of annotated junctions. Table 1 lists all gene annotations used to determine our set of annotated junctions. We froze these annotations on 24 January 2016 and compressed them into an archive available at http://verve.webfactional.com/misc/jan_24_2016_annotations.tar.gz. We ran the script https://github.com/nellore/runs/blob/master/sra/rip_annotated_junctions.py with PyPy v2.5.0 to extract junctions from these annotations, performing coordinate conversions from *hg38* to *hg19* where appropriate. The final list of junctions we defined as “annotated” is available at https://github.com/nellore/runs/blob/master/sra/annotated_junctions.tsv.gz.

### Selecting human SRA samples

Samples were selected by querying the SRA metadata SQLite database of the R/Bioconductor package SRAdb [9]. The database was downloaded from http://gbnci.abcc.ncifcrf.gov/backup/SRAmetadb.sqlite.gz, but this file is updated regularly. The version of SRAmetadb.sqlite.gz we used was updated on 1 April 2015, and it is available at ftp://ftp.ccb.jhu.edu/pub/langmead/sra_junctions/SRAmetadb.sqlite.gz. We selected all run_accessions from the sra table with platform = ’ILLUMINA’, library_strategy = ’RNA-Seq’, and taxon_id = 9606 (human) that also had URLs for FASTQs on the European Bioinformatics Institute server listed in the fastq table. Our query may be reproduced with the script https://github.com/nellore/runs/blob/master/sra/define_and_get_fields_SRA.R compatible with R v3.1.0.

### Alignment of human SRA samples with Rail-RNA

Rail-RNA v0.1.7b [7] was used for alignment. We aligned to *hg19* rather than the more recent *hg38* assembly because of *hg19*’s continued prevalence, including use by the GEUVADIS consortium [37] in its study of 462 lymphoblastoid cell line (LCL) samples as well as the GTEx consortium [38] in its ongoing large-scale study of gene expression across human tissues. We performed a single pass of alignment; that is, reads were not realigned after junctions were discovered to improve alignments of short-anchored reads. See the “Junction detection” subsection below. Alignment was performed in the cloud using AWS Elastic MapReduce on Elastic Compute Cloud spot instances, i.e., standardized units of computing capacity. Spot instances permit bidding for computing to save money, where bids that equal or exceed a market price are fulfilled. However, if the market price drops below a bid, instances could be lost, and a computational job could fail. So saving money by bidding for spot instances comes with risk, and rather than aligning all samples in one batch, we distributed this risk by dividing alignment up into 43 batches of about 500 samples each. Analysis of each batch was itself divided into (1) a preprocessing job flow, which downloaded and preprocessed compressed FASTQs from the European Bioinformatics Institute’s mirror of the SRA, writing results to Amazon’s cloud storage service S3; and (2) an alignment job flow, which was configured to write only exon-exon junction coordinates and the number of reads in each sample mapping across each detected junction. Each preprocessing job flow was run on a cluster of 21 c3.2xlarge instances, each with 8 Intel Xeon E5-2680 v2 (Ivy Bridge) processing cores and 15 GB of RAM. Each alignment job flow was run on a cluster of 61 c3.8xlarge instances, with 32 Intel Xeon E5-2680 v2 (Ivy Bridge) processing cores and 60 GB of RAM. Summing the sizes of the 43 compressed files output by the 43 runs gives 5.3 GB, about the size of an alignment BAM for a single RNA-seq sample. Our alignment runs may be reproduced by following the instructions at https://github.com/nellore/runs/blob/master/sra/README.md.

### Alignment cost

Alignment was performed over a period of eight days. There were 21,506 samples spanning 62.2 trillion nucleotides initially selected for alignment, but two samples (run accession numbers SRR651690 and DRR023700) were not found on the European Bioinformatics Institute server and were therefore excluded. We used the Amazon Cost Explorer to compute total cost; summing across eight days of activity, it came to US$15,393.69, or 72 cents per sample. Costs divided up by Amazon service over the period of computational activity may be viewed at https://github.com/nellore/runs/blob/master/sra/hg19.costs.csv.

### Junction detection

Rail-RNA’s junction detection method, discussed in detail in the Rail-RNA study [7], begins by using Bowtie 2 [39] in local alignment mode (–local) to align each read to the genome. If a read’s highest scoring alignment is soft-clipped, the read is retained and used for junction discovery. Otherwise, it is not used for junction discovery, on the principle that the parsimonious explanation for the read is that it is exonic. Reads with soft-clipping are then divided into short, overlapping segments called readlets. Readlets are aligned to the reference genome, and the alignments are clustered into sets of mutually compatible alignments. A gap between consecutively aligning readlets in a cluster is called as an exon-exon junction if an appropriate two-base motif (e.g., GT and AG) appears on either side of the corresponding intron in the reference. If multiple clusters are tied for largest, indicating an ambiguously mapped read, Rail-RNA refrains from using that read for junction discovery.

Rail-RNA’s approach is both parsimonious, seeking to explain alignments with as few junctions as possible, and conservative, ignoring evidence from multi-mappers. Accordingly, for this study, we value precision over recall in order to make reliable statements about junctions missed by annotation. The approach could underestimate (1) the number of reads mapping across a junction in a sample, and (2) the number of samples in which a given junction is found. Since Rail-RNA excludes reads that align to the genome end-to-end from its junction discovery algorithm, it is also liable to miss junctions in a given gene for which there is a processed pseudogene. Details on Rail-RNA’s single-pass alignment algorithm may be found in Sections S.18 and S.19 of the Rail-RNA study [7].

### Reproducing main figures

All data underlying Figs. 1, 2, 3, 5, and 6 are reproducible with the Python v2.7 script https://github.com/nellore/runs/blob/master/sra/tables.py, which was run using PyPy v2.5.0. These figures as well as Fig. 4 were generated with the Mathematica v10.3.1 notebook; see https://github.com/nellore/runs/blob/master/sra/preprint_figures.nb. SEQC/MAQC-III consortium junction data were downloaded from http://www.nature.com/nbt/journal/v32/n9/extref/nbt.2957-S4.zip. BioSample submission dates for 77 SRA runs (0.3% of the samples we studied) were not found on the server, so these runs were excluded from the analyses involving junction discovery dates presented in Figs. 5 and 6.

### Analysis of TRAP-seq samples

All 15 mouse TRAP-seq samples were taken from the study SRP031883; individual run accession numbers are provided in the Rail-RNA manifest file https://github.com/nellore/runs/blob/master/sra/translatome.manifest. These samples were aligned to *mm10* on a local computer cluster with Rail-RNA v0.2.3b, and junction output available at https://github.com/nellore/runs/blob/master/sra/mm10_translatome_junctions.tsv.gz may be recovered with the script https://github.com/nellore/runs/blob/master/sra/translatome.sh. Junctions were subsequently lifted over to *hg19* with the UCSC liftOver utility [36] using the command-line parameters -ends 2 -minmatch=1.0; that is, we lifted over only the two-base motifs on either end of each intron and required that all four motif bases had mappings in the liftover. The script https://github.com/nellore/runs/blob/master/sra/translatome.py calls the liftOver utility and writes lifted-over junctions and their presence in human annotation. Lifted-over junctions may be downloaded at https://github.com/nellore/runs/blob/master/sra/translatome_mm10_to_hg19_junctions.tsv.gz, where the format of this file is described in translatome.py. Statistics on the presence of lifted-over junctions in human SRA samples reported in the main text were computed by https://github.com/nellore/runs/blob/master/sra/get_final_translatome_stats.sh.

### Analysis of novel *ALK* isoform

The junction inclusion ratio *D* discussed in the main text is defined as follows. Suppose the number of instances where junctions are overlapped by reads (i.e., the junction overlap count) in *ALK* exons 1–19 is *A*, and the junction overlap count in *ALK* exons 20–29 is *B*. The normalized difference *D*=(*B*−*A*)/(*A*+*B*) is close to 1 when exons 1–19 are unexpressed compared to exons 20–29, and close to -1 when exons 20–29 are unexpressed compared to exons 1–19.

The *ALK* analysis may be reproduced by first filtering intropolis for junctions in *ALK* with the script https://github.com/nellore/runs/blob/master/sra/alk.sh, and then running the Mathematica 10.3.1 notebook https://github.com/nellore/runs/blob/master/sra/alk.nb. Samples found were checked manually for their descriptions on the SRA at http://www.ncbi.nlm.nih.gov/sra, and the UCSC Genome Browser screenshot of Fig. 7 was created using the Genome Browser’s PDF/PS utility.

### Principal component analysis

Restrict attention to unannotated junctions found in at least 1000 of the 21,504 SRA samples we studied and further to only those samples with at least 100,000 reads each. Consider the number of reads *c*_*ij*_ overlapping the *i*th unannotated junction in the *j*th sample. We formed the normalized log-counts $x_{ij}:=log_{2}\left (\frac {c_{ij}} {C_{j}}+1\right)$, where *C*_*j*_ is the number of mapped reads for sample *j*. We then used the row-centered matrix *A* for PCA; that is, $A_{ij} = x_{ij} - \bar {x}_{i}$. More specifically, we computed the cross product *A* ^*t*^ *A* in a block-wise manner, and we subsequently performed a singular value decomposition (SVD) of *A* ^*t*^ *A* to obtain the right-singular vectors (principal components) with a randomized SVD algorithm [40]. Three correlates of PC1 are mentioned in the text. They are defined as

$$\begin{array}{*{20}l} s_{j} &= \sum_{i} x_{ij}  \\ \ell_{j} &= \log_{2}(1+p_{j})  \\ m_{j} &= \log_{2}(1+C_{j})\,,  \end{array} $$

where *j* indexes samples and *p*_*j*_ is the read length in sample *j*.

Scripts for reproducing the PCA analysis are available in the sra subdirectory of https://github.com/nellore/runs and described in https://github.com/nellore/runs/blob/master/sra/README.md. The output of the analysis sourced the Mathematica 10.3.1 notebook https://github.com/nellore/runs/blob/master/sra/preprint_figures.nb for generating Fig. 4.

### Liftover of intropolis

http://intropolis.rail.bio also provides a version of intropolis with junction coordinates lifted over from *hg19* to *hg38*. This was accomplished with the UCSC liftOver utility [36] using command-line parameters -ends 2 -minmatch=1.0; that is, we lifted over only the two-base motifs on either end of each intron and required that all four motif bases had mappings in the liftover, as in the TRAP-seq analysis. The script https://github.com/nellore/runs/blob/master/sra/liftover_intropolis.py reproduces our liftover.
